# A rare case of invasive necrotizing myositis of the thigh caused by *Basidiobolus ranarum*: a multi-pronged approach to successfully managing a near-fatal polybacterial mycotic infection

**DOI:** 10.22034/cmm.2025.345248.1614

**Published:** 2025-08-03

**Authors:** Dhira Shobith Munipati, Navin Sundar Arunachalam Jeykumar, Balamourougan Krishnaraj, Venkatesh Arumuga Nainar, Anitha Gunalan, Rakesh Singh, Sanjay Sriram*S, Nanda Kishore Maroju

**Affiliations:** 1 Department of Surgery, Jawaharlal Institute of Postgraduate Medical Education and Research, Pondicherry, India; 2 Department of Microbiology, Jawaharlal Institute of Postgraduate Medical Education and Research, Pondicherry, India; 3 Department of Pathology, Jawaharlal Institute of Postgraduate Medical Education and Research, Pondicherry, India

**Keywords:** Antifungal agents, Basidiobolomycosis, *Basidiobolus ranarum*, Potassium iodide

## Abstract

**Background and Purpose:**

Traumatic fungal diseases are relatively less common and present significant challenges in treatment. In some cases, there is progressive spread and deep soft tissue colonization, especially in immunocompromised patients and those showing neglect and non-compliance with treatment. This pattern is common in patients from rural settings who are unaware of the consequences of delaying medical care and the resulting complications.

**Case Report:**

This study reported a case of Basidiobolomycosis manifesting as deep necrotizing myositis of the left thigh complicated by secondary bacterial sepsis in a 46-year-old immunocompetent man. *Basidiobolus ranarum*, was morphologically identified, isolated in culture and supported by wet mount microscopy and histopathology. It was treated with a multipronged strategy due to a refractory infection showing an unsatisfactory response to fungal monotherapy.

**Conclusion:**

The diagnosis was evasive due to the clinical picture of overt soft-tissue necrosis resembling a highly virulent bacterial infection showing antibiotic resistance. Broad aseptate hyphae in potassium hydroxide mount (KOH 10%) preparation led us to suspect the Entomophthorales organism and initiate prompt antifungal chemotherapy.

## Introduction

Basidiobolomycosis is a rare fungal infection that can affect the skin as a subcutaneous slow-growing mass, involve the gastrointestinal tract, and rarely result in systemic disease [ 1 ]. *Basidiobolus* species are thermophilic fungi commonly found on the skin, and especially the intestinal tracts of insects or animals in warm and humid areas. They are only occasional human pathogens, typically transmitted by insect bites [ 2 ]. They thrive in soil, decaying vegetation, and faeces of amphibians and reptiles, and infection also happens through direct inoculation by trauma [ 3 ]. This study aimed to report, perhaps, the first case of necrotising deep soft-tissue colonization caused by *Basidiobolus ranarum*, complicated by superimposed bacterial infection culminating in limb amputation.

## Case Report

This study reported the case of a 46-year-old man who presented at JIPMER, Puducherry, India, in 2023 with a chronic, non-healing ulcer over the posterior aspect of the left thigh for two years. The ulcer had started after an unknown insect bite, which was neglected and eventually increased in size. The patient had sought irregular treatment at local hospitals, where he had the wound cleaned and dressed along with oral empirical antibiotics. He did not have co-morbid illnesses or any immunocompromised conditions. 

**Figure 1:**
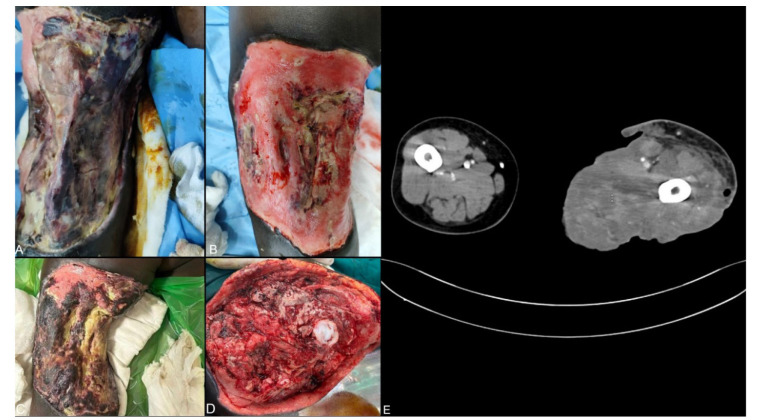
necrotic patch with underlying unhealthy granulation. Figure 1B. One week after admission: necrotic muscles covered by granulation tissue. Figure 1C. Two weeks after admission: underlying extensive myonecrosis after debridement. Figure 1D. Multiple abscess cavities and areas of myonecrosis observed intraoperatively. Figure 1E. Unviable posterior compartment muscles with normal contrast opacification of superficial femoral artery.

At the time of admission, the ulcer had spread to involve the thigh posteriorly, medially, and laterally, sparing only the anterior aspect. The floor had necrotic muscle interspersed with unhealthy, infected granulation tissue with foul-smelling sero-purulent discharge (Figure 1A). He had unexplained foot drop on the involved side. All the distal lower limb pulses were palpable.

His haemoglobin level was 6.5 gm/dl, total leucocyte counts were 26,000 cells/µL (normal range: 4,000-11,000 cells/µL), and C-reactive protein peaked at 12.7 mg/dL (normal range: 0.3 to 1.0 mg/dL), and his vital signs were normal. He subsequently underwent wound debridement under regional anaesthesia and had an O-negative blood transfusion. A bacterial culture from a wound swab grew *Enterococcus faecalis*, *Escherichia coli*, and Proteus mirabilis, and the patient was started on Meropenem and Gentamicin injections. Over the next few days, the wound showed little improvement despite daily wound debridement under local anaesthesia (Figure 1B). Subsequently, a KOH 10% mount from the wound discharge showed broad aseptate hyphae. Although either Entomophthorales or Mucorales was possible, clinical conditions were more suggestive of mucormycosis; hence, intravenous amphotericin B was started. A computed tomography-angiogram showed normal contrast opacification of lower limb vessels with non-enhancing areas of the posterior and medial compartment muscles suggestive of non-viability (Figure 1E).

A week later, wound healing was still poor (Figure 1C), and the patient had daily fever spikes (average temperature of 39.6 °C), tachycardia (110/min), and persistent leucocytosis (25,300 cells/µL). A second wound swab again revealed polymicrobial infection with *Klebsiella pneumoniae*, *Proteus mirabilis*, and *Escherichia coli*. He was given intravenous Piperacillin-Tazobactam and Colistin. Blood culture was positive for *Escherichia coli* but negative for fungi.

A high trans-femoral amputation was planned considering local and systemic sepsis, and non-functional status of the limb. Intra-operatively, there was extensive myonecrosis of the posterior and medial compartment muscles with multiple abscess cavities indicating superimposed bacterial infection (Figure 1D). 

**Figure 2:**
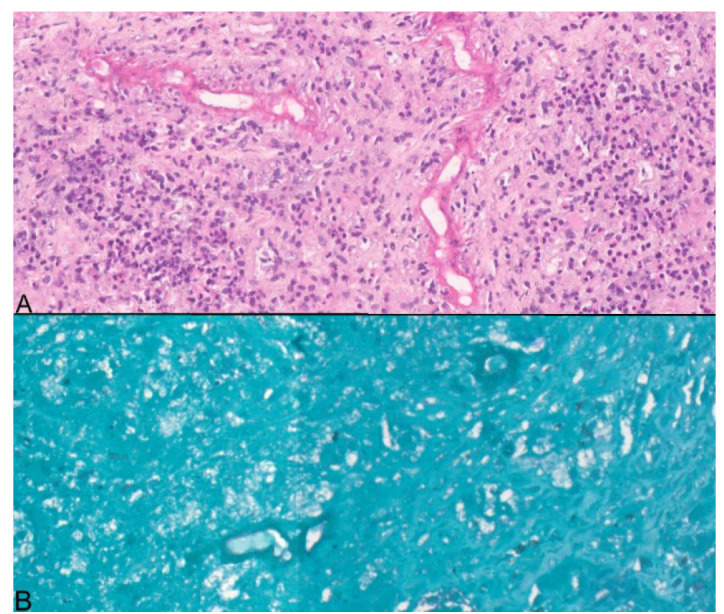
A. Irregular, broad hyphae with thin walls and rare septations, surrounded by eosinophilic granular material, Hematoxylin and Eosin stain, 20X magnification. Figure 2B. Hyphae highlighted by Gomori methenamine silver stain, 40X magnification.

Histopathology of tissue sections with hematoxylin and eosin staining showed dense inflammatory infiltrate forming micro-abscesses with foreign body giant cells and areas of necrosis. Scattered among them were broad, irregular fungal hyphae with thin wall and rare septa surrounded by eosinophilic granules (Splendore-Hoeppli phenomenon). There was no evidence of angioinvasion. This suggested *Basidiobolus ranarum* (Figure 2A). The fungal elements were also identified by periodic acid-Schiff and Gomori methenamine silver stains (Figure 2B). 

**Figure 3:**
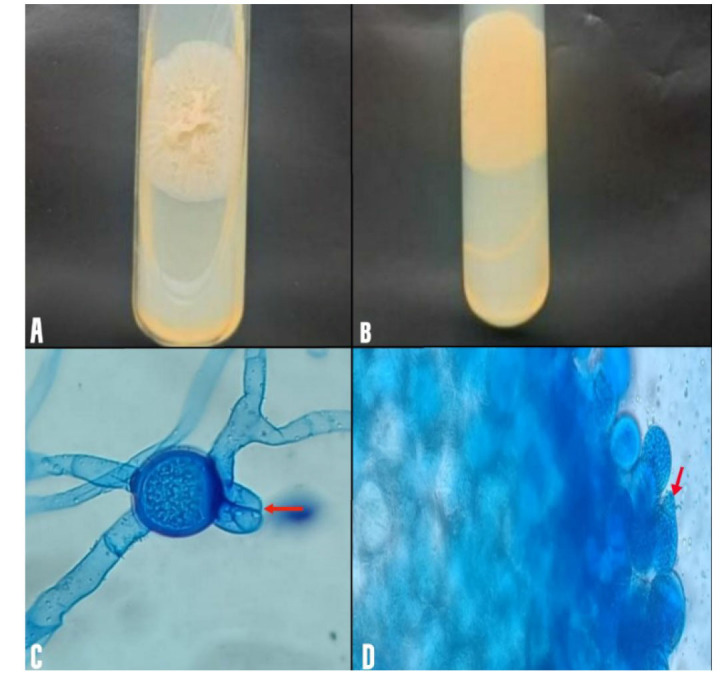
A. Obverse view showing heaped up and radially folded buff-to-grey colonies. Figure 3B. Reverse view of the culture shows buff color. Figure 3C. Wide hyphae and prominent beak-like appendage on one side (red arrow). Figure 3D. Ballistospore discharging a single-celled spore (red arrow).

The KOH 10% mount from the sample showed broad aseptate hyphae. The sample was inoculated into two Sabouraud dextrose agar (SDA) tubes. One was incubated at 25 °C and another at 37°C. After 5 days of incubation at 37 °C, the SDA agar tube showed thin, flat, waxy, buff-to-grey colonies which were heaped up and radially folded; the reverse side was buff-colored and non-pigmented (Figure 3A and 3B). Lactophenol cotton blue tease mount preparation was made from the culture, which showed many round intercalary zygospores (20-50 µm) with smooth, thick walls and prominent beak-like appendage on one side, which was identified as *Basidiobolus ranarum* (Figure 3C). The fungi had wide hyphae having occasional septa, and short sporophores were seen, which enlarged apically to form a swollen area from which single-celled spores and fragments of sporophore are forcibly discharged, known as ballistospores (Figure 3D). *Mycobacterium tuberculosis* infection was ruled out by both liquid culture (Mycobacteria Growth Indicator Tube 960 or MGIT 960) and cartridge-based nucleic acid amplification test (CB-NAAT).

**Figure 4:**
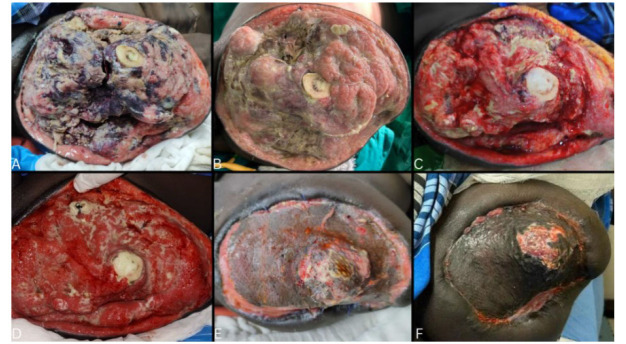
A to D. Progressive improvement of the wound over nearly 6 weeks. Figure 4E. Post-operative day 5 of split skin grafting. Figure 4F. Follow-up after 3 months of oral antifungal therapy.

Despite aggressive therapy, stump infection progressed with ascending myonecrosis (Figure 4A). Wound swab culture grew *Enterococcus faecium*, *Escherichia coli*, and *Pseudomonas aeruginosa* with overlapping sensitivities to Linezolid and Cefoperazone-Sulbactam. On the 6^th^ post-operative day, the patient developed septic shock with tachycardia (125/min), hypotension (BP- 90/60 mm of Hg), fever (40.2 °C), and delirium. He was started on noradrenaline infusion. Blood cultures were positive for *Pseudomonas aeruginosa*, and intravenous Ceftazidime was added. Amphotericin B was stopped, and intravenous voriconazole (200 mg twice daily) was added after morphological identification of *Basidiobolus ranarum* by culture. Voriconazole was chosen due to poor clinical response to the amphotericin B, unavailability of *in vitro* susceptibility results, and lack of guidelines for the management of Basidiobolomycosis. Voriconazole has also been shown to be highly effective in rare fungal infections or infections showing resistance to other antifungal agents [ 4 ]. The patient was also started on oral Lugol’s iodine (5% elemental iodine and 10% potassium iodide) empirically, starting at 10 drops orally twice a day and increased gradually to a maximum of 20 drops over 2 weeks. Lugol’s iodine was considered based on several case reports confirming its successful use in deep soft tissue Basidiobolus infections and the possibility of intrinsic resistance to azoles. Although the general condition of the patient improved over the next five days, considerable wound improvement took nearly four weeks. Voriconazole was stopped on the ninth day, but oral Lugol’s iodine was continued for 6 weeks. The wound was cleansed with normal saline, and tissue debris was removed twice daily. He also underwent periodic wound debridement under regional anaesthesia over the next four weeks for removal of necrotic tissue and control of local sepsis. Thyroid function test was performed at four weeks and found to be within acceptable limits. With this protracted, multipronged approach, the wound granulated and contracted over the next six weeks (Figures 4 B-D).

**Figure 5:**
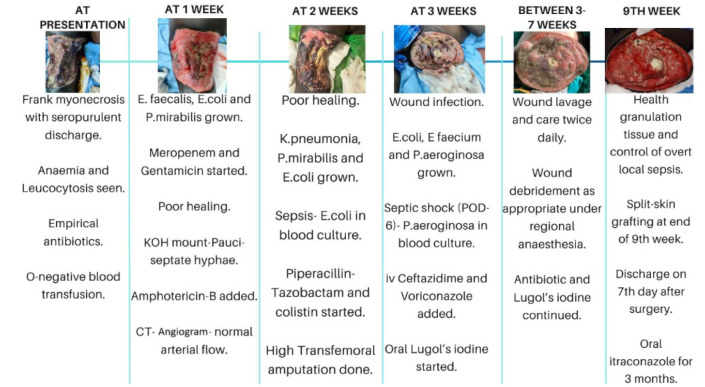
Timeline of disease progression and management.

The patient underwent split-skin grafting and was discharged on postoperative day 7 (Figure 4E). Considering the severity of infection, the patient was asked to take oral itraconazole (200 mg twice daily) for three months to ensure local eradication of infection (Figure 4F). The wound has completely healed since then, and the patient remains on three-monthly follow-up visits. The timeline of progression and management is represented in Figure 5.

## Discussion

Zygomycosis refers to any infection caused by fungi (zygomycetes) in the phylum Zygomycota, the only fungi known to infect humans. Zygomycota includes 10 major orders, among which Mucorales and Entomophthorales contain species that can infect humans. The order Entomophthorales includes the genera *Basidiobolus* and *Conidiobolus*. However, contemporary phylogenetic investigations of fungi formerly classified within the outdated phylum Zygomycota have led to the reclassification of the zoopathogenic genera *Basidiobolus* and *Conidiobolus* as members of the newly established monophyletic phylum Entomophthoromycota. The Entomophthoromycota is organized into three classes, namely Basidiobolomycetes (*Basidiobolus* spp.), Neozygitomycetes (*Neozygitis* spp.), and Entomophthoromycetes (*Conidiobolus* spp., *Completoria* spp., *Entomophthora* spp., and others). According to these taxonomic and phylogenetic revisions, the genus *Basidiobolus* is now situated within the class Basidiobolomycetes, order Basidiobolales, and family Basidiobolaceae, while the genus *Conidiobolus* is located within the class Entomophthoromycetes, order Entomophthorales, and family Ancylistaceae [ 5 ].

*Basidiobolus* species are commonly associated with subcutaneous lesions of the thigh, buttock, and trunk, with involvement of the gastrointestinal tract also reported [ 6 ]. *Basidiobolus ranarum* was first isolated from frogs in 1886 and later discovered in mammals, such as dogs, bats, horses, and humans, with horses and humans considered traditional mammalian hosts. Despite the ubiquitous distribution of the fungus, most human diseases have been reported in the tropical and subtropical regions, especially Indonesia, Africa, South America, and Southeast Asia [ 7 ]. 

Mode of infection is believed to occur by implantation of spores of the organism via minor trauma, such as insect bites, thorn prick, and rubbing contaminated leaves after defecation on the perineum or by inhalation of spores [ 6 , 8 , 9 ]. It is more common in children with a male preponderance [ 9 ]. While Mucorales is responsible for “opportunistic infections” in immunocompromised hosts, *Conidiobolus* and *Basidiobolus* infections occur primarily in immunocompetent hosts [ 7 ]. There are eight phylogenetically distinct species of Basidiobolus, which include *B. haptosporus*, *B. heterosporus*, *B. magnus*, *B. meristosporus*, *B. microspores*, *B. minor*, *B. omanensis*, and *B. ranarum* [ 10 ]. 

Recent genomic analysis, focusing on allelic variation in *B. ranarum*, suggests a diploid nuclear genome with an estimated size of 700 Mb. The research also identified redundancy in elongation factors, specifically overlapping paralogs of EF-1a. The researchers hypothesize that genome duplication plays a significant role in maintaining overlapping genes within *B. ranarum* [ 11 ].

Usually, the disease starts as a hard, non-tender nodule which may enlarge and rarely ulcerate [ 2 , 12 , 13 ], but does not disseminate [ 14 ]. Although deep invasion is uncommon, subcutaneous disease can be invasive locally and penetrate into the adjacent fat, muscle, fascia, and even bone [ 15 ]. *Basidiobolus ranarum* produces enzymes, like lipase and protease, that probably produce the clinical manifestations of infection [ 2 ].

Wound swabs or tissue bits (as in our case) are inoculated on Sabouraud’s agar and show visible growth 2 to 3 days after incubation at 25-30 °C with typical pale yellow or white colonies with radial folds. Lactophenol cotton blue wet mount shows large, broad vegetative hyphae, and thick-walled zygospores with beak-like appendages. On staining with Grocott-Gomori methenamine silver or Periodic acid Schiff, the organism appears as thin-walled, broad, and aseptate fungal hyphae [ 12 ]. In haematoxylin and eosin staining, they show the Splendore-Hoeppli phenomenon–an epithelioid cell granuloma (multinucleated giant cell) with longitudinal and transversal 4- 12 mm *B. ranarum* hyphae accompanied by eosinophilic infiltration [ 2 , 3 , 12 ]. 

Angioinvasion is rare, in contrast to Mucorales, which can cause vascular thrombosis [ 7 ]. If culture results are negative, molecular methods, like DNA probes targeting the 18S ribosomal subunit, DNA sequencing with panfungal primers, or real-time polymerase chain reaction targeting the cytochrome b gene, are used to confirm the genus and species [ 16 ]. Serodiagnosis with immunodiffusion can be employed as an adjunct method of diagnosis; the test appears to be very specific for *Basidiobolus ranarum* and exhibits no cross-reactivity with other species of the order Entomophthorales [ 17 ]. This test is useful for monitoring infected patients and diagnosis when the culture is negative for growth [ 18 ]. 

No single drug has proved effective in the treatment of Basidiobolomycosis, and mixed results have been reported with therapies, including potassium iodide, amphotericin B, ketoconazole, fluconazole, itraconazole, trimethoprim-sulfamethoxazole, and surgery [ 8 , 18 ]. Resistance to amphotericin B has been reported in more than 50% of cases [ 18 ]. Some studies have reported spontaneous resolution occasionally in localised subcutaneous zygomycosis, but surgical excision is not advised as it fails to resolve local colonization and might even extend the spread of infection [ 8 , 13 , 18 ]. While favourable responses with fluconazole, posaconazole, and itraconazole have been observed with low minimal inhibitory concentrations, voriconazole has also been used successfully for Basidiobolomycosis [ 19 ]. With some studies showing intrinsic resistance to azoles, other studies have shown good outcomes with oral potassium iodide, resulting in complete local resolution of infection [ 13 , 18 , 19 , 20 ].

## Conclusion

Although subcutaneous *Basidiobolus* infection has been reported in the literature, a frank necrotising infection of this severity in an immunocompetent patient is unprecedented so far. It is critical to have a higher index of suspicion for fungal infection when there is failure to respond to antibiotics. This has been a peculiar case in which a multipronged approach involving dual systemic antifungal agents, antibiotics, and multiple surgeries had to be employed to subdue the ascending infection, considering its worrying proximity to the pelvis. In our observation, oral potassium iodide proved decisive, and subsequently, the infection was eradicated with three months of oral itraconazole. However, it should be noted that earlier medical care and timely intervention would have saved the limb of the patient.
